# Good Laboratory Practices Are Not Synonymous with Good Scientific Practices, Accurate Reporting, or Valid Data

**DOI:** 10.1289/ehp.0901495

**Published:** 2010-02

**Authors:** Frederick S. vom Saal, John Peterson Myers

**Affiliations:** University of Missouri, Columbia, Missouri, E-mail: vomsaalf@missouri.edu; Environmental Health Sciences, Charlottesville, Virginia

In her commentary, Tyl (2009) responded to our criticism (Myers et al. 2009) of her bisphenol A (BPA) research (Tyl et al. 2008), and she defended the reliance on Good Laboratory Practices (GLP) in animal studies concerning risks posed by chemicals. Her commentary, however, provides additional evidence that her research on BPA is flawed and that GLP can be unreliable.

The key evidence can be found in her treatment of the effect of BPA on prostate weight (Tyl et al. 2008). This effect is important because Tyl’s data on adult prostate in mice has been used by the chemical industry—which has funded all of Tyl’s research on BPA—and the Food and Drug Administration (FDA) to conclude that BPA has no effect at low doses. Indeed, Tyl argued that the weight of the evidence supports her findings that BPA is safe (all industry-funded studies report no low-dose effects of BPA). In contrast, > 200 studies in experimental animals, all funded by government agencies, have reported significant effects of BPA at low doses that are relevant to human and ecologic exposures (vom Saal et al. 2007).

We (Myers et al. 2009) concluded that prostate weights reported by Tyl et al. (2008) were abnormally high in control males, suggesting either that the dissections were done improperly, that control animals were exposed to a contaminating estrogen, or that their prostates were diseased. This would render the results invalid and therefore inappropriate to use in assessing BPA safety. It would also provide insights as to why, despite many other studies showing adverse effects of exposure to BPA at low doses (vom Saal et al. 2007), Tyl et al. (2008) detected none.

To counter this criticism, Tyl (2009) presented a table (her Table 2) of mouse prostate weights from other laboratories. The data she presented in fact show that no other laboratory measuring prostate weight in mice has reported mean weights as high as those reported by Tyl et al. (2008), except in old male mice with diseased prostates. Tyl’s table cites data from research published by Heindel et al. (1995) previously conducted at her own institution, Research Triangle Institute, although she did not acknowledge this. The mean prostate weight reported by Heindel et al. (1995) for 16- to 17-week-old CD-1 male mice was 48 mg, which is similar to most other findings, but contrasts sharply with Tyl’s mean prostate weights of 74 mg for the F_1_ males in her BPA study (these males were identified in Table 1 of Tyl’s commentary as being examined at 18 weeks of age).

Table 2 of Tyl (2009) also includes data from a publication by Morrissey et al. (1988) showing a mean prostate weight of 58 mg in 23-week-old CD-1 mice. However, this study involved comparing data from two laboratories, and Tyl omitted from her table the data from the second laboratory that reported a mean prostate weight of 35 mg in 23-week-old CD-1 males. Morrissey et al. (1988) observed that the laboratory reporting the mean of 58 mg also had a higher standard deviation and lower statistical sensitivity than the laboratory reporting the 35 mg mean prostate weight. In studies in which prostate weight is high, such as that of Tyl et al. (2008), the findings are suspect in that the abnormally high prostate weight data show a poor relationship to other male reproductive organs (Morrissey et al. 1988). This strongly suggests that nonprostatic tissue has been included when prostate weights are abnormally high in the absence of disease.

Tyl’s discussion of prostate weight effects also suggests that studies identified as GLP may not adhere to the strict record-keeping goals to which GLP aspires, undermining one of the arguments used for the value of GLP over research funding by the National Institutes of Health, which rarely follows the costly GLP guidelines. In the original publication, Tyl et al. (2008) reported that F_1_ retained males were necropsied at approximately 14 weeks of age. In Table 1 of her commentary (Tyl 2009), Tyl stated that these males were 18 weeks of age at necropsy. However, in testimony before the FDA Science Board BPA Subcommittee hearing on 16 September 2008 (FDALive.com 2008), Tyl stated that these males were 24 weeks of age at necropsy as an explanation for their high prostate weights. Tyl assured the FDA panel that since “the difference in age influences growth rate and growth of organs, the comparison [of 12- and 24-week-old males] is specious, it is comparing apples and oranges.” In fact, Tyl’s data in Table 1 of her commentary (Tyl 2009) show no relationship between age and body weight. The inconsistencies in Tyl’s FDA testimony, which could have had a significant impact on a regulatory decision concerning BPA, and the data concerning the age at tissue collection, prostate weights, and body weights presented in Table 1 of her commentary are disturbing, and indicate that a thorough review of original data in Tyl et al. (2008) by scientific experts is warranted.
